# Monoderm bacteria: the new frontier for type IV pilus biology

**DOI:** 10.1111/mmi.14397

**Published:** 2019-10-08

**Authors:** Vladimir Pelicic

**Affiliations:** ^1^ MRC Centre for Molecular Bacteriology and Infection Imperial College London London UK

## Abstract

In the diverse world of bacterial pili, type IV pili (Tfp) are unique for two reasons: their multifunctionality and ubiquity. This latter feature offers an extraordinary possibility, that is, to perform comparative studies in evolutionarily distant species in order to improve our fragmentary understanding of Tfp biology. Regrettably, such potential has remained largely untapped, because, for 20 years, Tfp have only been characterised in diderm bacteria. However, recent studies of Tfp in monoderms have started closing the gap, revealing many interesting commonalities and a few significant differences, extending the frontiers of knowledge of Tfp biology. Here, I review the current state of the art of the Tfp field in monoderm bacteria and discuss resulting implications for our general understanding of the assembly and function of these widespread filamentous nanomachines.

## Graphical abstract

Type IV pili (Tfp) have been studied for decades, primarily because they are widespread weapons in pathogenic bacteria. An in‐depth understanding of how these filamentous nanomachines are assembled and function, which is yet to be achieved, could have important consequences for human health. Here, I review the current state of the art of the field in bacteria with one membrane, which have only recently emerged as an advantageous model for studying Tfp biology.

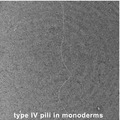

## Tfp at a glance

Pili are hair‐like filamentous polymers composed of proteins generically named pilins, found at the surface of most prokaryotes (Bacteria and Archaea). Multiple unrelated pilus types have been defined, which are composed of different types of pilins and are assembled by distinct protein machineries (Hospenthal *et al.*, 2017). As revealed by three decades of studies, Tfp display a set of unique and highly distinctive features (Berry and Pelicic, 2015).

**Figure 1:**
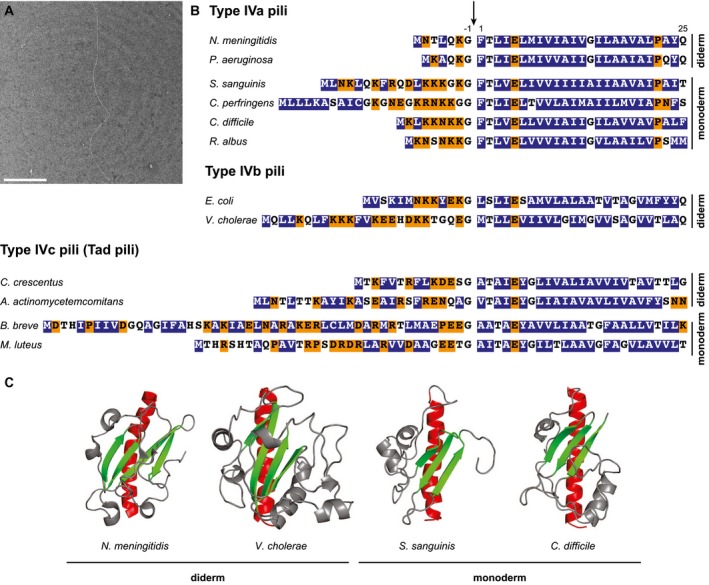
Tfp and their major pilin subunits in monoderms. A. Tfp morphology in *S. sanguinis* as assessed by transmission EM after negative staining. Scale bar represents 500 nm. B. Sequence alignment of class III signal peptides in major pilins in selected monoderm and diderm bacteria. Hydrophilic residues are shaded in orange, hydrophobic residues in blue, and neutral residues are unshaded. The site of processing by the prepilin peptidase is indicated by a vertical arrow. C. 3D structures of major pilins in monoderm and diderm bacteria. Conserved structural features have been highlighted including the C‐terminal part of the α1 helix in red and the anti‐parallel β‐sheet in green. *Nota bene*: the protruding N‐terminus of the α1 helix (α1N), which corresponds to the long stretch of hydrophobic residues in the class III signal peptide and gives full‐length pilins their typical ‘lollipop’ 3D structure, is not represented here because it is often truncated in structural studies to improve protein solubility. The PDB entries that were used to generate this figure are: 5JW8 (*N. meningitidis*), 1OQV (*V. cholerae*), 6I2O (*S. sanguinis*) and 4PE2 (*C. difficile*).

First, Tfp share both morphological and molecular characteristics, which allow their easy identification. In brief, Tfp are (i) flexible filaments, 4‐8 nm in diameter and several µm long (Fig. 1A), with often a propensity to form bundles, and (ii) polymers of predominantly one major type IV pilin, assembled by conserved multiprotein machineries. Type IV pilins (hereafter simply named pilins) share an N‐terminal sequence motif known as class III signal peptide (Fig. 1B) and a typical ‘lollipop’ 3D structure (Giltner *et al.*, 2012). The N‐terminal motif (IPR012902 entry in the InterPro database) (Jones *et al.*, 2014) consists of a hydrophilic leader peptide (6‐51 residues long) ending with a Gly, followed by a tract of 21 mostly hydrophobic residues, except for a negatively charged Glu_5_ (Fig. 1B). This hydrophobic tract, which is part of an extended N‐terminal α‐helix (α1N) of about 50 residues, represents the ‘stick’ of the lollipop protruding from a globular head (Giltner *et al.*, 2012). The globular head consists of the C‐terminal half of α1 packed against a β‐sheet made of several antiparallel β‐strands (Fig. 1C). Besides pilins, many other proteins involved in Tfp biology are also very distinctive (Berry and Pelicic, 2015) including an aspartic acid protease named prepilin peptidase (IPR000045), a platform protein at the base of the assembly machinery (IPR003004), a hexameric filament assembly (extension) ATPase (IPR007831), *etc*.

The second defining feature of Tfp is that they are exquisitely multifunctional and have been associated with an array of seemingly unrelated functions, which is likely to expand even further with the characterisation of Tfp from seldom studied species of Bacteria and Archaea. Properties associated with Tfp include (i) adhesion to a wide variety of biotic and abiotic surfaces, (ii) the formation of cell aggregates, (iii) a form of surface‐associated motility known as twitching motility, (iv) DNA uptake during natural transformation *etc*. It should be noted, however, that no Tfp‐expressing species display all these properties, some of which are more common than others.

The third defining feature is that although Tfp have been studied only in a handful of diderm bacteria for 20 years, mostly Proteobacteria, genes encoding pilus assembly proteins are actually ubiquitous in bacterial and archaeal genomes (Imam *et al.*, 2011; Berry and Pelicic, 2015). However, not all the corresponding proteins are involved in the assembly of *bona fide* Tfp. Rather, they assemble a variety of filamentous nanomachines – type 2 secretion systems (T2SS), competence pseudopili, and archaella – which are evolutionarily related to Tfp, hence their grouping in a super‐family named type IV filaments (Tff) (Berry and Pelicic, 2015). However, although there is often some overlap, these nanomachines can be readily distinguished from Tfp because they have different morphological characteristics and/or mediate different properties. For example, T2SS form short pseudopili mediating protein secretion in diderm bacteria (Korotkov and Sandkvist, 2019).

## Monoderm bacteria produce Tfp

In this review, I will focus exclusively on *bona fide* Tfp in monoderm bacteria (hereafter simply referred to as monoderms), the first description of which was in *Ruminococcus albus*, a cellulolytic species of Firmicutes from the order of Clostridiales. The ability of *R. albus* to adhere to cellulose was found to be mediated by Tfp (Rakotoarivonina *et al.*, 2002). High‐resolution negative staining electron microscopy (EM) micrographs revealed thin, long, flexible and moderately bundling pili on the surface of *R. albus*, which were shown by immuno‐EM to be composed of GP25 – a pilin with a canonical class III signal peptide (Fig. 1B) (Rakotoarivonina *et al.*, 2002).

Tfp were subsequently found to be widespread in other species of Clostridiales, including in important human pathogens *Clostridium perfringens* and *Clostridioides difficile* (formerly known as *Clostridium difficile*). *C. perfringens* was found to produce short filaments mediating twitching motility (although it was originally referred to as gliding motility), which were absent in a mutant in the platform protein (Varga *et al.*, 2006). In *C. difficile*, high‐resolution EM and immuno‐EM micrographs confirmed that filaments in Clostridiaceae display classical Tfp morphology and are primarily composed of PilA1, a pilin with canonical class III signal peptide (Fig. 1B) and 3D structure (Fig. 1C) (Piepenbrink *et al.*, 2014; 2015).

The last Firmicutes in which Tfp have been described is *Streptococcus sanguinis*, a common cause of infective endocarditis in humans. Tfp are not found in other *Streptococcus* species as they are encoded by a locus found only in *S. sanguinis* (Gurung *et al.*, 2016), probably acquired by horizontal transfer from an unknown donor. *S. sanguinis* Tfp, which have a Tfp morphology (Fig. 1A), were purified to homogeneity and shown to be heteropolymers of two similar pilins (PilE1 and PilE2) in a 4:3 ratio (Berry *et al.*, 2019). These pilins exhibit canonical class III signal peptide (Fig. 1B) and 3D structure (Fig. 1C) (Berry *et al.*, 2019). It remains to be seen whether such a heteropolymeric structure might be a more general property of Tfp in monoderms, which awaits purification and biochemical characterisation of filaments in other species. However, it is now clear that the classical definition that Tfp are always homopolymers composed of one major pilin must be moderated.

Firmicutes are not the only phylum of monoderms in which Tfp have been studied. *Bifidobacterium breve*, a probiotic species of the phylum Actinobacteria, was found to express filaments composed of a canonical pilin (Fig. 1B), as shown by immuno‐EM (O'Connell Motherway *et al.*, 2011). In addition, piliation was abolished in a mutant in the extension of ATPase. Tfp have also been identified in another Actinobacteria, *Micrococcus luteus*, where they are involved in natural transformation as shown by a systematic genetic analysis (Angelov *et al.*, 2015). Visualisation of *M. luteus* filaments was difficult, but they could be enriched after shearing off the bacterial surface and were shown to be composed primarily of the major pilin Flp (Fig. 1B).

## Tfp biogenesis machineries in monoderm bacteria: the beauty of simplicity

Early studies have identified two different sub‐types of Tfp, named Tfpa and Tfpb, based on sequence features of their major pilin and genetic organisation of the genes involved in filament assembly (Pelicic, 2008; Giltner *et al.*, 2012). In brief, Tfpa were defined as consisting of smaller pilins with shorter leader peptides, with Tfp assembly genes scattered around the genome. In contrast, Tfpb consist of larger pilins with longer leader peptides, with assembly genes clustering together. While it is now clear that these defining features are obsolete (see below), phylogenetic studies have confirmed the validity of this sub‐division by showing that Tfpa and Tfpb are phylogenetically distinct systems (Kachlany *et al.*, 2001; Denise *et al.*, 2019). Moreover, this revealed that Tad pili, which were previously thought to be a sub‐family of Tfpb (Tomich *et al.*, 2007), actually constitute a distinct clade and should therefore be renamed Tfpc. A global survey (Denise *et al.*, 2019) revealed that Tfpa and Tfpc display a wider taxonomic distribution in Bacteria than Tfpb, which are found only in Proteobacteria. Interestingly, Tfpa and Tfpc are likely to be expressed by hundreds of bacterial species belonging to all major phyla of monoderms (*e.g.* Actinobacteria, Firmicutes, Thermotogae, Chloroflexi, *etc*.) (Denise *et al.*, 2019). Consistent with this, Tfp studied so far in monoderms belong to the Tfpa and Tfpc sub‐types.

**Figure 2:**
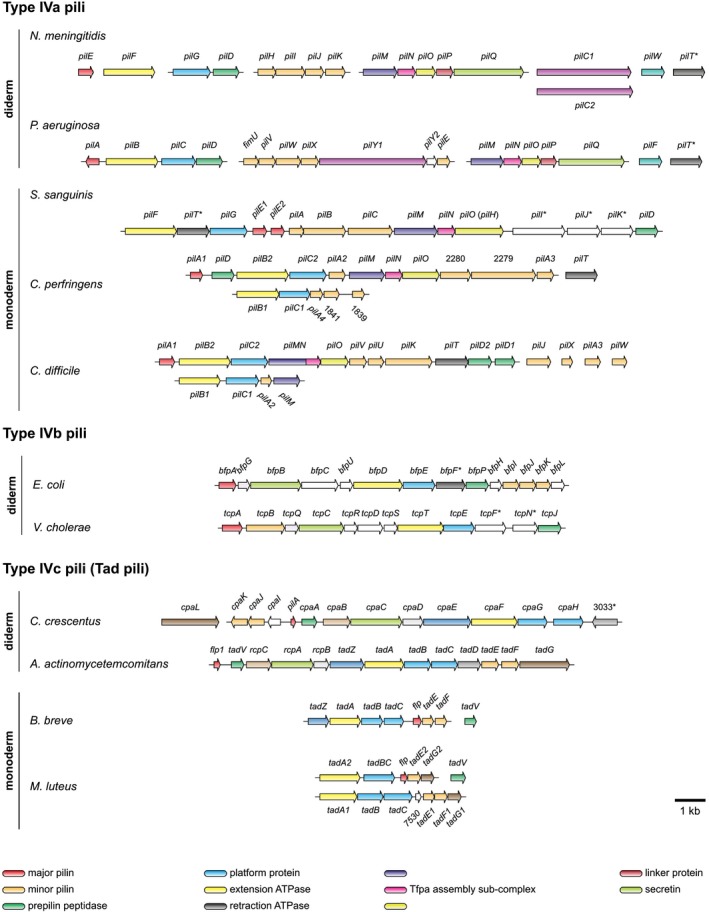
Genes involved in Tfp biogenesis in monoderms. All the genes are drawn to scale, with the scale bar representing 1 kb. Genes shaded in the same colour are likely to encode orthologous proteins based on sequence similarity, functional domain conservation and/or conserved synteny. Names of genes often mentioned in this review are listed at the bottom. No colour coding indicates genes with no clear homologs in other species. Genes with no name are identified by their ORF number in the corresponding genome, genes highlighted with an * have been shown to encode proteins dispensable for Tfp biogenesis but playing key roles in Tfp biology. The former nomenclature for the *pilO* gene in *S. sanguinis* is indicated within parentheses. The major pilin in *C. perfringens* has not been experimentally determined and has been attributed to *pilA1* based on findings in *C. difficile*. The NCBI entries that were used to generate this figure are: PRJEA34687 (*N. meningitidis*), PRJNA331 (*P. aeruginosa*), PRJEB7884 (*S. sanguinis*), PRJNA79 (*C. perfringens*), PRJNA78 (*C. difficile*), PRJNA15630 (*E. coli*), PRJNA36 (*V. cholerae*), PRJNA32027 (*C. crescentus*), PRJNA40107 (*A. actinomycetemcomitans*), PRJNA13487 (*B. breve*) and PRJNA238160 (*M. luteus*).

Tfp studied so far in Firmicutes are clearly Tfpa, although their major pilins have long leader peptides (Fig 1B) and their Tfp assembly genes cluster together (Fig. 2). Indeed, these Tfp are (i) composed of pilins with a α1N highly similar to that of major pilins in diderm Tfpa models (Fig. 1B), (ii) assembled by machineries that contain the PilM (IPR005883), PilN (IPR007813) and PilO (IPR007445) proteins (Fig. 2) specific of Tfpa (Denise *et al.*, 2019) and (iii) retractile filaments whose retraction is powered by the retraction ATPase PilT (IPR006321). A detailed comparison of Tfpa biogenesis genes in monoderms and diderms reveals striking overall conservation (Fig. 2), but also a few interesting differences. In the diderm models, *Neisseria meningitidis* and *Pseudomonas aeruginosa*, Tfpa biogenesis machineries are virtually identical. Systematic mutagenesis studies have shown that filament biogenesis requires 15‐17 proteins (Pelicic, 2008): a pilin, several minor (low abundance) pilins (four in *N. meningitidis*, five in *P. aeruginosa*), a prepilin peptidase, a platform protein, an extension ATPase, an assembly sub‐complex (PilM, PilN and PilO), a secretin pore (PilQ) in the outer membrane (OM), a linker (PilP) between the assembly sub‐complex and secretin, an OM lipoprotein stabilising PilQ (PilW in *N. meningitidis*) and an OM protein involved in the late stages of Tfp biogenesis (PilC1/PilC2 in *N. meningitidis*). Filament retraction is powered by PilT, which is dispensable for piliation (Craig *et al.*, 2019). In *S. sanguinis*, the only monoderm in which a systematic genetic analysis of piliation has been performed, filament biogenesis relies on a very similar machinery, even more than we previously appreciated (Gurung *et al.*, 2016). Indeed, the use of a sensitive protein homology detection tool (HHpred) capable of identifying remote homology (Zimmermann *et al.*, 2018) reveals that *pilH* actually encodes a protein showing N‐terminal similarity to PilO. I therefore propose to rename this gene *pilO* to reflect this important finding, and to be consistent with the general nomenclature. Filament biogenesis in *S. sanguinis* requires ‘only’ 10 proteins (Gurung *et al.*, 2016): one major pilin (PilE1 or PilE2), three minor pilins (PilA, PilB and PilC), a prepilin peptidase, a platform protein, an extension ATPase and an assembly sub‐complex (PilM, PilN and PilO). Strikingly, all the components involved in late stages of Tfpa biogenesis in diderms (such as crossing of the OM), that most often localise to the OM, are missing in monoderms which do not have a second membrane. As in diderms, filament retraction is powered by the retraction of ATPase PilT, which is dispensable for piliation (Gurung *et al.*, 2016). It is important to underline that the term ‘minor pilin’ is just generic, only indicating that the corresponding proteins are cleaved by the prepilin peptidase to become low abundance components of the filaments (Berry *et al.*, 2019). This does not imply that these proteins play identical or even similar functions in each system, which is supported by the fact that minor pilins in different systems share no sequence homology. For example, PilA, PilB and PilC in *S. sanguinis* are unrelated to PilH, PilI, PilJ and PilK in *N. meningitidis*. However, findings in *S. sanguinis* are expected to be broadly applicable to Tfpa in other monoderms, such as Clostridiaceae, where all the above genes are conserved (Melville and Craig, 2013; Maldarelli *et al.*, 2014), with only minor differences (Fig. 2). Another important conclusion is that the molecular mechanisms of Tfpa assembly and retraction are likely to be the same in monoderms and diderms.

Tfp studied in Actinobacteria are clearly Tfpc. Indeed, they are composed of pilins (i) with the signature Flp motif (Kachlany *et al.*, 2001) identified in the α1N of Tfpc major pilins (Fig. 1B), (ii) with a very small size (44 residues in *B. breve*, 68 residues in *M. luteus*) and (iii) that are assembled by machineries that contain two paralogs of the platform protein (Fig. 2) specific of Tfpc (Denise *et al.*, 2019). A detailed comparison of Tfpc biogenesis genes in monoderms and diderms reveals some conservation (Fig. 2) and significant differences. In the diderm models, *Caulobacter crescentus* and *Aggregatibacter actinomycetemcomitans*, Tfpc machineries are virtually identical (Tomich *et al.*, 2007). Systematic mutagenesis studies have shown that filament biogenesis requires 13 proteins: a pilin, two minor pilins, a prepilin peptidase, two paralogous platform proteins, an extension ATPase, a secretin and several Tfpc‐specific proteins of unknown function (RcpB, RcpC, TadD, TadG and TadZ) (the *A. actinomycetemcomitans* nomenclature is used here for the sake of clarity). In monoderms, the simplification trend discussed above for Tfpa is also obvious for Tfpc and is even more extreme. In *B. breve*, the Tfpc machinery apparently consists only of a pilin, two minor pilins, a prepilin peptidase (encoded in a different locus), two paralogous platform proteins, an extension ATPase and TadZ (O'Connell Motherway *et al.*, 2011). Most of these genes are conserved in *M. luteus*, where there is extensive gene duplication, except for *tadZ* that is curiously ‘replaced’ by *tadG* (Fig. 2). A systematic genetic analysis of Tfpc biogenesis in *M. luteus* revealed that all the genes from both loci are essential for piliation suggesting that paralogs have non‐redundant roles (Angelov *et al.*, 2015). As in the case of Tfpa, the striking simplicity when compared to diderm models can be explained by the fact that all the components in diderms likely to be involved at late stages of Tfpc biogenesis (*e.g.* the secretin) and/or localising to the OM (RcpB, RcpC and TadD) are ‘missing’ in monoderms.

Taken together, the simplicity of Tfpa and Tfpc biogenesis machineries in monoderms suggests that bacteria with one membrane could be advantageous to unravel how Tfp are assembled, which remains one of the most important open questions in the field.

## Tfp assembly: lessons from monoderm bacteria

What have studies of Tfp in monoderms revealed about filament assembly? In the case of Tfpa, which have been extensively studied in diderm models, the lessons are many and potentially far‐reaching.

First, since PilM, PilN and PilO proteins are found in monoderms and are essential for Tfpa biogenesis, they do not merely constitute an ‘alignment complex’ as they are often referred to in the literature (Craig *et al.*, 2019; McCallum *et al.*, 2019), whose role would be to connect the pilus assembly sub‐complex to the secretin in the OM, simply because there is neither secretin, nor OM in monoderms. Instead, these three proteins are involved in Tfpa assembly and are part of the assembly sub‐complex together with the platform protein and extension ATPase.

Second, unlike in diderms where PilN and PilO are similar in size and structure (their globular domains exhibiting a ferredoxin‐like fold) (McCallum *et al.*, 2019), PilO in monoderms is actually much larger than PilN (56.6 vs. 22.3 kDa in *S. sanguinis*) and shows similarity to PilO only in its N‐terminal portion. This suggests that it is a bifunctional protein with an unknown second role unrelated to filament assembly. A fascinating possibility, although entirely speculative at this stage, is that PilO in monoderms might be involved in the translocation of the filaments across the thick layer of peptidoglycan (PG), and/or anchoring of the pilus assembly machinery to the cell wall.

Third, the set of proteins essential for Tfp biogenesis in *S. sanguinis* strikingly overlaps with the set of *N. meningitidis* proteins found to be involved in filament assembly *per se* (major pilin, prepilin peptidase, platform protein, extension ATPase, assembly sub‐complex PilM, PilN, PilO and PilP) (Carbonnelle *et al.*, 2006; Goosens *et al.*, 2017). The only apparent differences concern PilP, which is absent in *S. sanguinis*, and the fact that minor pilins are not part of the minimal synthetic machinery necessary for Tfp assembly in a non‐native heterologous host (Goosens *et al.*, 2017). There are suitable explanations for both these differences. Since PilP is the linker between the assembly sub‐complex and the secretin (Tammam *et al.*, 2013), its absence in monoderms where there is no secretin is logical. PilP requirement in a minimal synthetic Tfp assembly machinery (Goosens *et al.*, 2017) is therefore likely to be only indirect, because PilP is essential for the stability of the PilN‐PilO complex in diderms (Georgiadou *et al.*, 2012). As for the minor pilins, it has been shown in diderms that they become dispensable for piliation when pilus retraction is abolished by a second mutation in *pilT* (Winther‐Larsen *et al.*, 2005; Carbonnelle *et al.*, 2006), which means that they are not involved in filament assembly *per se*. It remains to be seen, however, if such a genetic suppression strategy would yield similar findings in monoderms.

**Figure 3:**
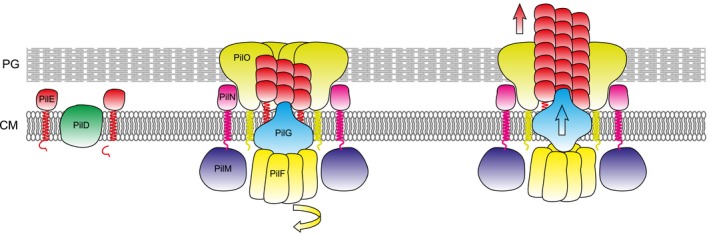
Molecular mechanisms of Tfpa assembly in monoderms: a working model. The model involves three steps, from left to right. The *S. sanguinis* nomenclature has been used here for the sake of clarity, and the same colour code than in Fig. 2 has been used. (1) Prepilins, which are translocated across the CM via the SRP‐Sec pathway, are processed by the prepilin peptidase PilD and constitute a reservoir of mature subunits, ready to be polymerised into filaments. (2) Filament assembly involves extrusion of the pilins from the CM and their polymerisation at the base of a growing filament, which is performed by a sub‐complex consisting of PilF‐PilG‐PilM‐PilN‐PilO. The topology of this sub‐complex is based on existing literature. ATP binding, hydrolysis and release drive a clockwise rotation of central sub‐pores in the PilF ATPase (indicated by the yellow arrow). (3) This rotatory movement thrusts the interacting PilG platform protein upwards (cyan arrow) and rotates it clockwise, before it falls back into the CM. This rotational motion pushes pilins out of the CM (red arrow) and allow their polymerisation in a right‐handed helical filament. Although the PilT retraction motor is not represented on this figure, it is thought that ATP binding, hydrolysis and release drive a counterclockwise rotation of PilT (hence reverse to PilF), which wrenches PilG downwards and facilitates PilE depolymerisation from the filament. CM, cytoplasmic membrane; PG, peptidoglycan.

Altogether, observations in monoderms and diderms suggest a hypothetical scenario for Tfpa assembly in monoderms (Fig. 3). Prepilins (major and minor) are first translocated via the SRP‐Sec pathway across the cytoplasmic membrane (CM) (Arts *et al.*, 2007; Francetic *et al.*, 2007), where they remain initially as bitopic proteins with their α1N behaving as a transmembrane domain. Their leader peptide is then processed after the conserved Gly by the prepilin peptidase (Aly *et al.*, 2013), which also methylates the first residue of mature pilins as confirmed in *S. sanguinis* (Berry *et al.*, 2019). It is unclear at this stage, if the minor pilins are assembled first and cap the filaments as proposed for other Tff (Korotkov and Hol, 2008). Next, filament assembly involves extrusion of the pilins from the CM and their polymerisation at the base of a growing filament, which is performed by a sub‐complex consisting of the platform protein, extension ATPase and PilM‐PilN‐PilO sub‐complex. Multiple findings, including cryo‐electron tomography (ET) of the entire machinery (Chang *et al.*, 2016), indicate that these components form a structure at the CM with several interconnected layers. The protein‐protein interactions are likely to be the same in all Tfpa systems (Fig. 3). In brief, PilM interacts with the N‐terminus of PilN (Georgiadou *et al.*, 2012), forming a ring at cytoplasmic side of the CM (Chang *et al.*, 2016). In monoderms, this interaction is further supported by the fact that PilM and PilN are fused in *C. difficile* (Fig. 2) (Melville and Craig, 2013), and it might involve a conserved C‐terminal RD(MILF)N(FYW)FS motif in PilN, which is different from the motif involved in the PilM‐PilN interaction in diderms (Georgiadou *et al.*, 2012). PilN then interacts with PilO (Georgiadou *et al.*, 2012), forming a ring on the periplasmic side of the CM (Chang *et al.*, 2016). As speculated above, PilO in monoderms might be a bifunctional protein involved in the translocation of the filaments across the PG and/or anchoring of the assembly machinery to the cell wall (Fig. 3). The two rings spanning the CM accommodate the platform protein (Chang *et al.*, 2016), which interacts with the hexameric extension ATPase (Goosens *et al.*, 2017). A recent structural study has suggested that cycles of ATP binding, hydrolysis and release drive a clockwise rotation of central sub‐pores in the ATPase hexamer, which thrusts the interacting platform protein upwards and rotates it clockwise, before it falls back in the CM (McCallum *et al.*, 2017). This rotational motion pushes pilins out of the CM for their subsequent polymerisation in a right‐handed helical filament (Fig. 3). Recent cryo‐EM reconstructions of several Tfp revealed that the filaments are right‐handed helical polymers of pilins held together by extensive hydrophobic interactions between their α1 helices, which run approximately parallel to each other within the filament core (Kolappan *et al.*, 2016; Wang *et al.*, 2017). Extensive sequence conservation in α1N (Fig. 1B) suggests that Tfpa in monoderms have a similar structure. Furthermore, conservation of the helix‐breaking Pro_22_ suggests that α1N helices will be partially melted between Ala_14_/Gly_14_ and Pro_22_ as in diderm filaments, which is thought to make Tfpa flexible and elastic (Kolappan *et al.*, 2016; Wang *et al.*, 2017).

Since Tfpc assembly is less understood (Tomich *et al.*, 2007), fewer lessons can be learned from monoderms but they are interesting nonetheless. It appears that Tfpc assembly in monoderms relies on a machinery much simpler than Tfpa. Actually, it might be one of the smallest, if not the smallest machinery capable of assembling canonical Tfp. Findings in monoderms suggest that the minimal machinery capable of assembling Tfpc consists of a major pilin and two minor pilins, which are processed by a prepilin peptidase, and polymerised by two paralogous platform proteins powered by an extension ATPase (O'Connell Motherway *et al.*, 2011; Angelov *et al.*, 2015). A CM protein is also involved, which is curiously different in *B. breve* (TadZ) and *M. luteus* (TadG). As a corollary, all the other Tfpc biogenesis proteins found in diderm models (Tomich *et al.*, 2007) are likely to act after filament assembly, possibly at the step during which filaments cross the periplasm and OM in order to emerge on the cell surface. An important question is how the assembly sub‐complex is held together in Tfpc systems, when compared to Tfpa. Can the Tfpc‐specific CM component (TadZ/TadG) ‘substitute’ for the PilM‐PilN‐PilO complex? Or has the Tfpc platform extension ATPase complex evolved to be stable and functional on its own, and if so how?

## Monoderm bacteria too display a wide array of Tfp‐mediated functions

Our mechanistic understanding of the many Tfp‐mediated functions remains incomplete, at best. Since many of these functions have also been reported for Tfp in monoderms, the study of these simpler filaments might illuminate some poorly understood mechanistic aspects.

Perhaps unsurprisingly, one of the first role attributed to Tfp in monoderms is adhesion to biotic/abiotic surfaces, a very common Tfp property and possibly the main reason why these filaments have been studied for decades (Berry and Pelicic, 2015). In contrast to other types of pili that usually harbour a minor pilin with intrinsic adhesive properties at their tip (Hospenthal *et al.*, 2017), it is not well understood how Tfp mediate adhesion. This could be clarified by further studying Tfpa and Tfpc in monoderms as there is growing evidence that both are ‘sticky’ filaments. Tfpc were found to be essential for efficient *in vivo* colonisation of the murine gut by *B. breve*, since a non‐piliated mutant was severely impaired in colonisation and persistence (O'Connell Motherway *et al.*, 2011). Strikingly, a minor pilin (TadE) was found to promote epithelial cell proliferation *in vivo*, suggesting that *B. breve* Tfp contribute to the maturation of the human gut in early life by stimulating growth of the epithelial mucosa (O'Connell Motherway *et al.*, 2019). For Tfpa, there is also ample evidence of their role in adhesion in a variety of monoderms. In *R. albus*, a cellulolytic species playing an important role in fibre breakdown in the rumen of herbivores, Tfp bind cellulose but it is not clear whether the major pilin GP25 has intrinsic binding activity or not (Pegden *et al.*, 1998; Rakotoarivonina *et al.*, 2002). In monoderms pathogenic for humans, Tfpa have been implicated in binding to host cells. Heterologous expression of *pilA2* from *C. perfringens* in a *pilE* non‐piliated mutant of *N. gonorrhoeae* was sufficient to restore piliation and confer attachment to mouse or rat muscle cell lines, to which the gonococcus does not normally adhere (Rodgers *et al.*, 2011). These findings directly implicate *C. perfringens* Tfpa, more specifically PilA2, in the ability of this species to adhere to muscle fibres, which is key to its pathogenic potential. Similarly, Tfpa were found to promote (i) the adherence of *C. difficile* to human intestinal epithelial cells and persistence in the intestine (McKee *et al.*, 2018), and (ii) the binding of *S. sanguinis* to human cells (Chen *et al.*, 2019). It is not known which pilus component mediates binding, but the presence of very large (>50 kDa) minor pilins with a ‘modular’ architecture, that is, with bulky C‐terminal functional domains, is certainly an intriguing possibility. In *S. sanguinis*, PilB and PilC contain C‐terminal domains often involved in adhesion in proteins unrelated to Tfp, a von Willebrand factor type A domain (IPR002035) and a concanavalin A‐like lectin/glucanase structural domain (SSF49899), respectively (Berry *et al.*, 2019). This suggests that *S. sanguinis* Tfp harbour two minor pilins with intrinsic adhesive properties, maybe at their tip, allowing the bacterium to bind host protein and carbohydrate ligands. In addition, in the above species, colonisation of host cells and surfaces is further enhanced by the ability of Tfpa to mediate the formation of cell aggregates (Bordeleau *et al.*, 2015), which eventually results in the formation of biofilms (Varga *et al.*, 2008; Maldarelli *et al.*, 2016; Chen *et al.*, 2019). It remains to be seen whether pilus‐pilus contacts leading to aggregation involve major or minor pilins.

Twitching motility, a distinctive property of Tfpa (Berry and Pelicic, 2015), was observed and studied in several monoderm Tfpa‐expressing species: *C. perfringens*, *C. difficile* and *S. sanguinis* (Varga *et al.*, 2006; Mendez *et al.*, 2008; Gurung *et al.*, 2016; Purcell *et al.*, 2016). Actually, studies in the 1970's, showing that most *S. sanguinis* isolates were capable of surface‐associated motility with the hallmarks of twitching motility, were the first evidence for the presence of Tfp in monoderms (Henriksen and Henrichsen, 1975). Twitching motility is a PilT‐powered process during which bacteria use Tfp as ‘grappling hooks’ to attach to a surface and then pull themselves upon filament retraction towards the site where the pilus is attached. Tensile forces generated upon PilT‐mediated pilus retraction were measured in *S. sanguinis* and found to be remarkably similar to those in Gram‐negative Tfpa‐expressing species (*i.e.* hundreds of pN), despite these bacteria having widely different surface architectures. A systematic genetic analysis identified four *S. sanguinis* proteins required for twitching motility but dispensable for pilus biogenesis (PilI, PilJ, PilK and PilT), the first three of which are not found in diderm Tfpa‐expressing species (Gurung *et al.*, 2016). This offers the unprecedented possibility to characterise twitching motility at an atomic level without interference of an OM.

The last property that was associated with Tfp in monoderms is DNA uptake during natural transformation. In most diderm competent species such as *N. meningitidis*, Tfpa bind‐free extracellular DNA and promote its import (or uptake) across the OM, upon PilT‐powered pilus retraction (Ellison *et al.*, 2018). DNA is then transferred to a second (Com) machinery that further translocates it across the PG and CM. In competent monoderm species, this early step in natural transformation is not mediated by Tfp but by a closely related Tff known as competence pseudopilus. This filamentous structure, which can be long or not, has been shown to bind DNA (Laurenceau *et al.*, 2013; Muschiol *et al.*, 2017). However, an unexpected twist comes from the analysis of DNA uptake in *M. luteus* that was found to be mediated by Tfpc (Angelov *et al.*, 2015), which were not previously known to be capable of mediating this property. This finding has important functional implications, especially since it suggests that Tfpc in *M. luteus* are retractile filaments capable of generating significant tensile force, which has recently been confirmed in diderms (Ellison *et al.*, 2017). Owing to its simple pilus biogenesis machinery (Fig. 2), *M. luteus* is therefore a promising model for unravelling the molecular mechanisms of PilT‐independent Tfpc retraction, which remain mysterious.

## Concluding remarks and future directions

Although the study of Tfp in monoderms is still in its infancy, it is now obvious that these filaments are not mere curiosities distantly related to Tfp in diderm models but *bona fide* Tfp, which might represent the long sought new research avenue capable of filling important gaps in our understanding of Tfp biology. The key advantage of monoderms is the simplicity of their Tfp biogenesis machineries, which stems from their simpler surface architecture. These new Tfp models, especially those with developed genetics like *S. sanguinis* and *M. luteus*, could help us to understand how filaments are assembled and function by performing in‐depth studies using a combination of genetics, two‐hybrid assays, cryo‐ET, structural biology, synthetic biology (the compact *S. sanguinis* system is a handy source of genes for reconstituting Tfpa into a non‐native monoderm host) *etc*.

Important advances (although this is obviously subjective) concerning the molecular mechanisms of filament biogenesis would be to (i) precisely define the roles of PilM, PilN and PilO proteins in Tfpa assembly, (ii) determine whether the minor pilins sit at the filament tip and prime filament assembly (Cisneros *et al.*, 2012; Nguyen *et al.*, 2015), (iii) understand how Tfp cross the mesh‐like PG layer (could the non‐canonical PilO protein be involved) and (iv) understand how the more rudimentary Tfpc machinery works. As for Tfp‐mediated functions, monoderms could prove key for understanding how (i) Tfp mediate adhesion by studying PilB and PilC modular pilins (this would have implications reaching far beyond adhesion since modular pilins with hundreds of different domain architectures are widespread), (ii) Tfpa retraction/extension dynamics orchestrate motility, possibly by using elegant labelling or label‐free strategies that allow dynamic filament visualisation (Ellison *et al.*, 2017; Tala *et al.*, 2019) and (iii) Tfpc retraction occurs in the absence of PilT.

In conclusion, recent advances in Tfp biology in monoderms – including Archaea that have been covered elsewhere (Chaudhury *et al.*, 2018) – outline a very exciting prospect for the years to come, that is, that monoderms might finally contribute to our mechanistic understanding of the assembly/function of Tfp and related filamentous nanomachines, which are ubiquitous in prokaryotes.
